# Perioperative selective decontamination of the digestive tract does not improve postoperative infectious complications after gastrectomy: a propensity score-matched analysis

**DOI:** 10.1007/s00423-026-03974-y

**Published:** 2026-01-22

**Authors:** Jasmin Hasanovic, Floris Berg, Christian Teske, Marius Distler, Jürgen Weitz, Daniel E. Stange, Felix Merboth

**Affiliations:** 1 Department of Visceral, Thoracic, and Vascular Surgery, University Hospital Carl Gustav Carus, Technische Universität Dresden, Fetscherstrasse 74, 01307 Dresden, Germany; 2 National Center for Tumor Diseases (NCT/UCC), Dresden, Germany; 3 German CancerResearch Center (DKFZ), Heidelberg, Germany; 4 University Hospital and Faculty of Medicine Carl Gustav Carus, Technische Universität Dresden, Dresden, Germany; 5 Helmholtz-Zentrum Dresden-Rossendorf (HZDR), Dresden, Germany

**Keywords:** Gastric cancer, Gastrectomy, Selective decontamination of the digestive tract, Surgical site infection, Anastomotic leak, Pneumonia, Propensity score matching

## Abstract

**Purpose:**

Infectious complications remain a significant cause of morbidity after gastrectomy for gastric cancer. Selective decontamination of the digestive tract (SDD) is effective in colorectal surgery, but its role in upper gastrointestinal procedures is unclear.

**Methods:**

We conducted a retrospective cohort study of patients undergoing open gastrectomy between 2014 and 2024. Patients treated with perioperative SDD (2018–2024) were compared with a matched historical cohort without SDD (2014–2018) using 1:1 propensity score matching (PSM). The primary endpoint was infectious complications.

**Results:**

A total of 108 patients were matched (54 SDD, 54 non-SDD). Rates of major complications (16.7% vs. 14.8%), anastomotic leakage (5.6% vs. 3.7%), superficial surgical site infections (SSIs) (14.8% vs. 11.1%), and organ/space infections (7.4% vs. 13.0%) were similar between the two groups. However, pneumonia occurred significantly more frequently in the SDD group (18.5% vs. 3.7%, *p* = 0.029). Microbiological analyses revealed more enterobacteriaceae and enterococci in the SDD group, with resistant isolates detected exclusively in this cohort.

**Conclusion:**

SDD did not reduce infectious complications after gastrectomy and was associated with higher pneumonia rates and the emergence of resistant isolates. These findings question its routine use in gastric cancer surgery and highlight the need for further prospective evaluation.

**Supplementary Information:**

The online version contains supplementary material available at 10.1007/s00423-026-03974-y.

## Introduction

Gastric cancer remains a major global health burden, ranking among the top five causes of cancer-related deaths worldwide, with more than one million new cases diagnosed annually [1]. Surgical resection with D2 (extended nodal dissection) lymphadenectomy is the cornerstone of curative treatment, often combined with neoadjuvant or perioperative chemotherapy [2]. Despite advances in surgical and perioperative care, postoperative morbidity following gastrectomy remains considerable, with complication rates reported between 10% and 20% in high-volume centers, depending on patient comorbidities, tumor stage, and surgical approach [3–7].

Infectious complications are not only the most common type of postoperative morbidity but also among the most impactful. These complications prolong hospital stay, increase healthcare costs, and contribute to early postoperative mortality. More importantly, postoperative infections, particularly major complications such as Clavien–Dindo (CD) grade ≥ IIIa, have been independently associated with impaired long-term oncologic outcomes in gastric cancer [8, 9]. Among these, pneumonia and organ/space infections are particularly relevant due to their systemic consequences, including increased morbidity and mortality risk, especially in elderly or comorbid patients [10–12].

Anastomotic leakage remains a serious complication after gastrectomy due to its association with severe infectious sequelae and elevated postoperative mortality [13]. Its occurrence is influenced by surgical technique, nutritional status, and microbiological factors, including bacterial translocation and gut flora composition [14–17].

Selective decontamination of the digestive tract (SDD) was developed to reduce postoperative infections by eliminating potentially pathogenic aerobic Gram-negative bacteria and yeasts from the oropharynx and gastrointestinal tract, while preserving anaerobic flora [16,18). In colorectal surgery, SDD has been consistently associated with reduced rates of anastomotic leakage and surgical site infections (SSIs) [18]. A recent meta-analysis demonstrated that SDD significantly reduces infectious complications in upper gastrointestinal surgery; however, robust evidence specific to gastrectomy remains limited [19]. Its role in pancreatic surgery is also less conclusive [20, 21]. Despite promising results in other surgical fields, evidence for SDD in gastric cancer surgery is limited, and potential adverse effects, such as microbiota disruption and the emergence of antimicrobial resistance, remain concerns.

In July 2018, our institution implemented perioperative SDD as part of a standardized protocol for upper gastrointestinal surgery. This study aimed to evaluate the effect of perioperative SDD on major complications (CDC grade ≥ IIIa), anastomotic leakage, superficial SSIs, organ/space infections, and pneumonia in patients undergoing open gastrectomy. Using propensity score matching (PSM) to reduce selection bias, we compared outcomes with a matched historical cohort treated prior to the introduction of SDD.

## Methods

### Study design and setting

This retrospective cohort study was conducted at the Department of Visceral, Thoracic, and Vascular Surgery, University Hospital Carl Gustav Carus Dresden, a high-volume tertiary referral center for gastrointestinal oncology. The study aimed to evaluate the effect of perioperative SDD on the rate and severity of postoperative infectious complications following open gastrectomy for gastric cancer. The study protocol was approved by the institutional review board (BO-EK-379082025), and all procedures were conducted in compliance with the ethical standards of the Declaration of Helsinki. A waiver was granted for informed consent.

## Patient eligibility and study periods

Eligible patients were adults (≥ 18 years) who underwent either open gastrectomy or open extended transhiatal gastrectomy with curative intent for histologically confirmed gastric adenocarcinoma between March 2014 and January 2024. A standard gastrectomy with D2 lymphadenectomy was performed. The duodenum was transected using an Endo-GIA stapler without additional oversewing of the staple line. Reconstruction was achieved via a Roux-en-Y configuration, with an end-to-side esophagojejunostomy constructed using a circular stapler. Patients were stratified into two groups depending on whether they received SDD. The intervention group consisted of patients who underwent surgery between July 2018 and January 2024, during which SDD was implemented at our institution as part of a standardized perioperative protocol. The control group comprised a historical cohort of patients who underwent surgery between March 2014 and July 2018, prior to the implementation of SDD. Exclusion criteria included minimally invasive or emergency procedures, palliative treatment, or incomplete perioperative or postoperative documentation necessary for outcome assessment. Both groups were followed for 90 days.

## Perioperative SDD protocol

The SDD protocol was standardized. The SDD solution consisted of two components administered together: a suspension containing colistin, tobramycin, and amphotericin B (prepared by the clinical pharmacy of the Carl Gustav Carus University Hospital) [22], and a vancomycin solution or capsules (off-label use). The suspension was prepared using 2.5 g of amphotericin B, 1 g of colistin, and 800 mg of tobramycin, while vancomycin was administered at a dose of 1 g and 20 mL of 0.9% sodium chloride. Patients scheduled for extended transhiatal gastrectomy underwent mechanical bowel preparation with 3 L of bowel irrigation solution the day before surgery. All patients received two doses of SDD on the evening before surgery. After creation of the esophagojejunostomy, the first local administration of SDD was performed intraoperatively in the area of the anastomosis via a gastric tube. Postoperatively, 4 doses were taken orally each day until the 5th postoperative day. If oral intake was not yet tolerated, SDD was administered via a nasogastric feeding tube.

## Standardized perioperative care

All patients in both groups received standardized perioperative care following institutional protocols. This included intravenous antibiotic prophylaxis with cefuroxime and metronidazole administered 30 to 60 min before incision, mechanical thromboprophylaxis, intensive pre- and postoperative respiratory physiotherapy, and early enteral feeding according to the Enhanced Recovery After Surgery (ERAS) protocol for gastrectomy [23]. Surgical resection techniques, extent of lymphadenectomy (D2), and reconstructive methods (e.g., Roux-en-Y reconstruction) were standardized across the study period and performed by experienced surgeons.

## Outcomes

The primary outcomes of this study were infectious complications, which were classified into three main categories: superficial SSIs; organ/space infections, including anastomotic leakage, duodenal stump insufficiency, and intra-abdominal abscesses; and other medical infections such as postoperative pneumonia or sepsis. Standard classification systems, including the Clavien-Dindo classification [24], were applied secondarily to ensure consistency in the assessment and reporting of complications.

For the definition of pneumonia, at least one major and two minor criteria had to be fulfilled (major: new infiltrate in chest imaging; minor: temperature > 38.5 °C or < 36.5 °C, new elevation or persistent high infection markers [leukocytes/C-reactive protein/procalcitonin], productive cough with sputum, pathogen detection) [25].

Secondary outcomes included overall morbidity, reoperation rates, sepsis, thromboembolic events, and mortality (both in-hospital and 30-day). To quantify the cumulative burden of postoperative morbidity, the Comprehensive Complication Index (CCI) was calculated for each patient. This index aggregates all postoperative complications into a single continuous metric weighted by severity.

### Microbiological analysis

Microbiological specimens were obtained according to the suspected site of infection. Sampling included wound swabs, puncture or drainage fluid, bronchial secretions or bronchoalveolar lavage, urine analysis, and blood cultures. All samples were processed in the institutional microbiology laboratory following standard protocols. Bacterial identification was performed using culture-based methods, supplemented by Gram staining. Pathogens were classified at the species level, and antimicrobial susceptibility was assessed by routine laboratory methods.

### Statistical analysis

Statistical analysis was performed using IBM SPSS Statistics (version 27). Continuous variables were presented as mean ± standard deviation (SD) or median with interquartile range (IQR), as appropriate. Group comparisons for continuous data were made using the independent-samples t-test or Mann–Whitney U test. Categorical variables were compared using the Chi-square test or Fisher’s exact test, depending on expected frequencies. All statistical tests were two-sided, and a p-value of less than 0.05 was considered to indicate statistical significance.

To minimize bias and improve comparability, PSM was performed using a 1:1 nearest-neighbor algorithm without replacement. The caliper width was set to 0.1. Propensity scores were calculated via logistic regression, including the following preoperative variables: age, sex, body mass index (BMI), American Society of Anesthesiologists (ASA) classification, smoking history, alcohol consumption, diabetes mellitus, neoadjuvant therapy (chemotherapy or radiochemotherapy), and type of gastrectomy. Covariate balance between matched groups was evaluated using standardized mean differences, graphically represented as a Love plot (Supplementray Table 1, Supplementary Fig. 1), and confirmed to be acceptable for all variables.

## Results

In the overall cohort, 141 patients were included, with 56 in the SDD group and 85 in the non-SDD group. Median age and BMI were similar between the groups, and the majority of patients were male and classified as ASA III. Rates of alcohol abuse, smoking, and comorbidities were comparable, although diabetes was more frequent in the SDD group. Preoperative treatment patterns were balanced, with slightly more neoadjuvant chemotherapy in the non-SDD group, and more patients without neoadjuvant therapy in the SDD group (Supplementary Table 2, Supplementary Table 3).

Following 1:1 PSM, a total of 108 patients were included in the final analysis, with 54 patients in each group: those who received perioperative SDD and those who did not (non-SDD). The matching process ensured that both cohorts were well balanced across all key baseline characteristics, allowing for a robust and unbiased comparison.

### Baseline and operative characteristics

Patient demographics and preoperative clinical variables demonstrated excellent comparability between the two groups (Table 1). The median age was 70.6 years (IQR 62.3–78.3) in the SDD group and 72.4 years (IQR 61.4–76.1) in the non-SDD group (*p* = 0.909). The proportion of male patients was slightly higher in the SDD group at 81.5% compared to 75.9% in the non-SDD group, although this difference did not reach statistical significance (*p* = 0.639). Other variables, such as BMI, ASA classification, smoking history, alcohol abuse, presence of diabetes mellitus, UICC stage and tumor localisation were evenly distributed between the cohorts. The use of neoadjuvant chemotherapy and radiochemotherapy was also comparable, ensuring homogeneity in pre-treatment oncologic burden.

**Table 1:** Patient characteristics

Variable	SDD (*n* = 54)	No SDD (*n* = 54)	*p*-value
Age, years (median, IQR)	70.6 (62.3–78.3)	72.4 (61.4–76.1)	0.909
Male gender (n, %)	44 (81.5)	41 (75.9)	0.639
BMI (median, IQR)	25.7 (22.5–28.3)	25.4 (22.9–28.7)	0.660
ASA Score			1.000
ASA II (n, %)	13 (24.1)	14 (25.9)	
ASA III (n, %)	37 (68.5)	37 (68.5)	
ASA IV (n,%)	4 (7.4)	3 (5.6)	
Alcohol use (n, %)	4 (7.4)	4 (7.4)	1.000
Smoking (n, %)	13 (24.1)	18 (33.3)	0.395
Diabetes mellitus (n, %)	22 (40.7)	19 (35.2)	0.692
Localisation of Tumor			0.119
Gastric carcinoma (n, %)	14 (25.9)	20 (37)	
Fundus (n, %)	2 (3.7)	4 (7.4)	
Corpus (n, %)	12 (22.2)	10 (18.5)	
Antrum (n, %)	0 (0)	6 (11.1)	
AEG (n, %)	40 (74.1)	34 (63)	
AEG Type II (n, %)	32 (59.3)	27 (50)	
AEG Type III (n, %)	8 (14.8)	7 (13)	
UICC Stage			0.788
UICC I (n, %)	14 (25.9)	12 (22.2)	
UICC II (n, %)	8 (14.8)	12 (22.2)	
UICC III (n, %)	17 (31.5)	17 (31.5)	
UICC IV (n, %)	15 (27.8)	13 (24.1)	
Neoadjuvant chemotherapy (n, %)	27 (50.0)	28 (51.9)	1.000
Neoadjuvant radiochemotherapy (n, %)	3 (5.6)	3 (5.6)	1.000
No neoadjuvant therapy (n, %)	24 (44.4)	23 (42.6)	1.000

Values are shown as n (%) unless otherwise indicated

SDD = selective decontamination of the digestive tract, AEG = Adenocarcinoma of the esophagogastric junction

Regarding operative details (Table 2), the types of surgical procedures performed were consistent between groups. Total gastrectomy was performed in 15 patients (27.8%) in the SDD group and 17 patients (31.5%) in the non-SDD group (*p* = 0.833), while the remaining patients underwent extended transhiatal gastrectomy. Rates of microscopically margin-negative (R0) resection were high in both groups, with 88.9% of SDD patients and 87.0% of non-SDD patients achieving microscopically negative margins (*p* = 1.000), indicating comparable oncologic completeness of resection. The median number of lymph nodes removed, an important surrogate for oncologic adequacy, was also similar, 24 nodes (IQR 19–32) in the SDD group and 24 nodes (IQR 20–29) in the non-SDD group (*p* = 0.656). Intraoperative blood loss was numerically lower in the SDD group (487.96 ± 285.5 mL) compared to the non-SDD group (602.08 ± 399.8 mL), though this difference did not reach statistical significance (*p* = 0.157).

**Table 2:** Operative characteristics

Variable	SDD (n = 54)	No SDD (n = 54)	*p*-value
Total gastrectomy (n, %)	15 (27.8)	17 (31.5)	0.833
Extended transhiatal gastrectomy (n, %)	39 (72.2)	37 (68.5)	0.833
R0 resection (n, %)	48 (88.9)	47 (87.0)	1.000
R1 resection (n, %)	6 (11.1)	7 (13.0)	1.000
Lymph nodes removed (median, IQR)	24 (19 – 32)	24 (20 – 29)	0.656
Positive lymph nodes (median, IQR)	1 (0 - 6)	1 (0 - 4)	0.794
Blood loss (mL, mean ± SD)	487.96 ± 285.5	602.08 ± 399.8	0.157

Values are shown as n (%) unless otherwise indicated

SDD = selective decontamination of the digestive tract

### Postoperative outcomes

Postoperative complications were classified and analyzed based on their severity and type, with a particular focus on infectious complications, including superficial SSIs, organ/space infections, and other infections such as pneumonia.

*Major postoperative complications*, defined as CD grade IIIa or higher, occurred in 9 patients (16.7%) in the SDD group and 8 patients (14.8%) in the non-SDD group (Table 3). This difference was not statistically significant (*p* = 1.000), indicating that SDD had no apparent effect on the incidence of life-threatening or intervention-requiring complications.

**Table 3:** Postoperative outcomes

Variable	SDD (*n* = 54)	No SDD (*n* = 54)	*p*-value
Overall morbidity (n, %)	28 (51.9)	33 (61.1)	0.438
Major complications CD grade ≥ IIIa (n, %)	9 (16.7)	8 (14.8)	1.000
Comprehensive Complication Index (mean ± SD)	19.45 ± 27.14	19.8 ± 25.52	0.698
Superficial SSIs (n, %)	8 (14.8)	6 (11.1)	0.776
Organ/space infections (n, %) Anastomotic leak (n, %) Duodenal stump leak (n, %)	8 (14.8) 3 (5.6) 2 (3.7)	8 (14.8) 2 (3.7) 2 (3.7)	1.000 1.000 1.000
Pneumonia (n, %)	10 (18.5)	2 (3.7)	**0.029**
Sepsis (n, %)	3 (5.6)	1 (1.9)	0.618
Length of hospital stay (day, median, IQR)	31 (17–36)	17 (10–20)	0.160
In-hospital mortality (n, %)	0 (0)	4 (7.4)	0.059
30-day mortality (n, %)	0 (0)	3 (5.6)	0.243

Values are shown as n (%) unless otherwise indicated

SDD = selective decontamination of the digestive tract; SSI = surgical site infection; CCI = Comprehensive Complication Index

*Superficial SSIs* were identified in 8 patients (14.8%) in the SDD cohort versus 6 patients (11.1%) in the control cohort (*p* = 0.776). While this complication occurred slightly more frequently in the SDD group, the difference was not statistically significant.

*Organ/space infections* occurred in 8 patients (14.8%) in both the SDD and non-SDD groups (*p* = 1.000), indicating no difference between the cohorts for this serious complication category. Among these, *anastomotic leakage* occurred in 3 patients (5.6%) in the SDD group and 2 patients (3.7%) in the non-SDD group (*p* = 1.000). *Duodenal stump leaks* occurred in 3.7% of patients in both the SDD and non-SDD groups, with no observed difference between them.

In contrast, *pneumonia* occurred significantly more often in patients who received SDD. This complication was observed in 10 patients (18.5%) in the SDD group versus only 2 patients (3.7%) in the non-SDD group, a difference that reached statistical significance (*p* = 0.029).

*Microbiological analyses* revealed that Gram-negative bacteria were the predominant isolates in both groups, accounting for 63.6% of isolates in the SDD group and 55.6% in non-SDD group, while Gram-positive bacteria represented 36.4% and 44.4%, respectively (Table 4). There was no significant difference in Gram-stain distribution between groups (*p* = 0.578). However, the overall distribution of specific pathogens differed significantly between groups (*p* = 0.025). Among Gram-negative organisms, Enterobacteriaceae were the most prevalent pathogens, isolated in 47.7% of SDD group and 27.8% of non-SDD group. Gram-negative rods other than Enterobacteriaceae were found at similar frequencies in both groups (13.6% vs. 22.2%). Among Gram-positive bacteria, Enterococci were more common in SDD group (25.0% vs. 5.6%), whereas Staphylococci and Streptococci were more frequently detected in non-SDD group (27.8% and 11.1%, respectively). Antibiotic susceptibility testing showed a significantly higher proportion of fully susceptible isolates in the non-SDD group compared to the SDD group (94.4% vs. 59.1%, *p* = 0.020). Intermediate susceptibility was more common in the SDD group (25.0% vs. 5.6%), and resistant isolates were exclusively detected in the SDD group (15.9% vs. 0%).

**Table 4:** Microbiological findings

Variable	SDD	No SDD	*p*-value
Sample location			0.338
Wound (n, %)	8 (18.2)	3 (16.7)	
Organ/space (n, %)	14 (31.8)	8 (44.4)	
Bronchial secretion (n, %)	9 (20.5)	0 (0)	
Urine (n, %)	9 (20.5)	5 (27.8)	
Blood (n, %)	4 (9.1)	2 (11.1)	
Gram stain			0.578
Gram-positive bacteria (n, %)	16 (36.4)	8 (44.4)	
Gram-negative bacteria (n, %)	28 (63.6)	10 (55.6)	
Pathogens			**0.025**
Gram-positive Staphylococci (n, %)	4 (9.1)	5 (27.8)	
Gram-positive Streptococci (n, %)	0	2 (11.1)	
Gram-positive Enterococci (n, %)	11 (25.0)	1 (5.6)	
Gram-negative Enterobacteriaceae (n, %)	21 (47.7)	5 (27.8)	
Other Gram-negative rods (n, %)	6 (13.6)	4 (22.2)	
Other bacteria (n, %)	2 (4.5)	1 (5.6)	
Antibiotic susceptibility			**0.020**
Susceptible (n, %)	26 (59.1)	17 (94.4)	
Intermediate (n, %)	11 (25.0)	1 (5.6)	
Resistant (n, %)	7 (15.9)	0 (0)	

Values are shown as n (%) unless otherwise indicated

SDD = selective decontamination of the digestive tract

*Postoperative antibiotics* were given to 22.2% of patients in the SDD group and 13% of patients in the non-SDD group (*p* = 0.206). Piperacillin/tazobactam was commonly used as first-line treatment. The median duration of postoperative antibiotic therapy was 15 days (IQR 7–25) in the SDD group and 8 days (IQR 7–10) in non-SDD group (*p* = 0.306) (Supplementary Table 4).

*Secondary outcomes* further supported these findings. Overall postoperative morbidity, defined as the presence of any complication regardless of severity, was observed in 28 patients (51.9%) in the SDD group and 33 patients (61.1%) in the non-SDD group (*p* = 0.438), suggesting no significant effect of SDD on general complication rates. The *CCI*, which quantifies cumulative postoperative morbidity on a continuous scale, was similar between groups (19.45 ± 27.14 in the SDD group vs. 19.8 ± 25.52 in the non-SDD group, *p* = 0.698), further indicating comparable overall burden of complications. The SDD group had a median postoperative hospital stay of 31 days (IQR 17–36), while the non-SDD group had 17 days (IQR 10–20) (*p* = 0.160).

*Mortality* revealed a numerical but statistically nonsignificant advantage for the SDD group. No patients in the SDD group died during their hospital stay or within 30 days postoperatively. In contrast, 4 patients (7.4%) in the non-SDD group died during their initial hospital admission, and 3 patients (5.6%) died within 30 days of surgery (*p* = 0.059 and *p* = 0.243, respectively).

Other complications, such as postoperative *intestinal paralysis*,* bleeding*,* thromboembolic events*, and *cardiac or renal complications*, occurred with similar frequency across groups and did not demonstrate any clear association with SDD administration.

In summary, perioperative SDD did not significantly influence rates of major complications, superficial SSIs, or organ/space infections including anastomotic leakage and duodenal stump leaks. However, it was associated with a statistically significant increase in the incidence of postoperative pneumonia. These findings question the utility of SDD in gastrectomy and suggest that its effect on respiratory infections may differ from its established role in colorectal surgery.

## Discussion

This study offers a detailed analysis of SDD effects in open gastrectomy for gastric cancer, focusing on key postoperative infectious outcomes. By using a propensity score-matched cohort design, we compared complication rates between patients with and without SDD. The results question the assumed universal benefit of SDD in upper gastrointestinal surgery and suggest its use in gastrectomy should be reconsidered.

The main finding of our analysis is that *pneumonia occurred significantly more frequently in the SDD group* than in the non-SDD group (18.5% vs. 3.7%, *p* = 0.029). This contrasts with previous studies in colorectal and pancreatic surgery, where SDD generally lowered infection rates, though its effect on pneumonia is less certain [16, 18]. A recent meta-analysis reported that SDD reduces pneumonia and anastomotic complications in upper gastrointestinal surgeries [19]. However, although SDD may reduce infectious complications in certain upper gastrointestinal procedures, the available studies are limited in number, often underpowered, and methodologically heterogeneous, making it difficult to draw definitive conclusions about its efficacy in gastric cancer surgery.

The higher pneumonia rates in the SDD group suggest a need to reconsider SDD’s mechanisms after upper gastrointestinal surgery. Though SDD aims to suppress pathogenic Gram-negative bacteria and yeasts, altering the gut and oropharyngeal microbiota may unintentionally weaken mucosal immunity and mucociliary clearance, raising susceptibility to respiratory pathogens. Studies showed microbiota disruption could harm alveolar macrophage function, compromise barrier integrity, and disrupt immune signaling, increasing risk of respiratory infections such as pneumonia [15, 17, 26].

The higher pneumonia rates may be due to the risks of open gastrectomy. Procedures like extended transhiatal resections involve manipulation near the diaphragm and lower mediastinum, which can temporarily impair breathing and increase pulmonary complication risk. Previous studies have demonstrated that total gastrectomy, prolonged operative time, and surgical trauma to the upper abdomen are significant risk factors for postoperative pneumonia [27]. This risk is further compounded in elderly patients and those with comorbidities such as impaired pulmonary function or diabetes [11, 27]. Additionally, alterations in normal esophagogastric anatomy and swallowing function following gastrectomy may promote microaspiration, particularly in frail or sarcopenic patients, contributing to pneumonia-related mortality [12]. Although these variables were accounted for by PSM, their interaction with SDD-induced microbiota disruption may have potentiated pulmonary vulnerability in our cohort.

In contrast to the significant difference observed in pneumonia, no statistically significant differences were found between groups in the other primary outcomes of interest, including major complications (CD grade > IIIa), anastomotic leakage, superficial SSIs, or organ/space infections. The rate of organ/space infections was identical in both groups (14.8%), indicating that perioperative SDD had no measurable effect on these severe infections. Anastomotic leakage rates were low in both groups (5.6% vs. 3.7%), aligning with published benchmarks and likely reflecting high surgical quality, rigorous perioperative care, and standardized anastomotic techniques at our center. Unlike colorectal studies where SDD reduced leaks and infections, our data showed no reduction in anastomotic leakage, superficial SSIs, or organ/space infections with SDD [18, 28, 29]. These findings suggest that SDD benefits may depend on procedure and site, as the upper gastrointestinal tract’s microbial environment and immune interactions differ from the colon [17]. Additional factors, such as exposure to oral flora, variations in anastomotic vascularization, and the anatomical and functional changes after gastrectomy, may also influence outcomes.

Interestingly, despite the administration of SDD, enterococci were significantly more prevalent in the SDD group, whereas staphylococci were more frequently isolated in non-SDD patients. This pattern suggests a selective shift in the microbial profile associated with SDD, potentially reflecting its targeted suppression of Gram-negative species while allowing certain Gram-positive organisms to persist or proliferate. However, these microbiological differences did not translate into a measurable reduction in overall infection rates. Similar shifts have been documented in intensive care unit settings, where SDD was associated with increased Enterococcus colonization despite no overall increase in infection rates [30]. The analysis of antibiotic susceptibility showed that the SDD group had fewer susceptible and more resistant isolates compared to the fully susceptible non-SDD group, supporting concerns that SDD can exert selective pressure favoring resistant microorganisms [31, 32].

Although in-hospital and 30-day mortality did not differ significantly between groups, it is noteworthy that there were no deaths in the SDD cohort, compared to 7.4% and 5.6% in the non-SDD cohort, respectively. While not statistically significant, this trend may suggest a potential benefit for certain subgroups, as seen in other studies [19]. However, due to the higher pneumonia rate in the SDD group, survival benefits should be interpreted with caution. Further prospective, larger-scale studies are necessary to clarify these observations.

This study has several strengths. The use of a matched cohort design allowed for balanced comparisons between treatment groups and minimized selection bias. The use of clearly defined and clinically relevant endpoints, major complications, anastomotic leakage, superficial SSIs, organ/space infections, and pneumonia, provides a nuanced picture of postoperative infectious morbidity. Furthermore, all patients were treated at a single high-volume academic center with consistent surgical and perioperative protocols, enhancing internal validity. However, important limitations must be acknowledged. First, the retrospective nature of the study imposes constraints on causal inference. Despite careful matching, residual confounding due to unmeasured variables, such as functional status, oral hygiene, pulmonary reserve, or specific microbiological profiles, cannot be ruled out. Second, the sample size, while sufficient to detect differences in pneumonia, may be underpowered for less frequent outcomes such as anastomotic leakage or mortality. Third, microbiological cultures and resistance patterns were not systematically collected from all patients, which limits insights into the biological effects of SDD.

Our findings indicate that SDD is not universally beneficial for all gastrointestinal procedures. While strong evidence supports its use in colorectal surgery, its impact on open gastrectomy is unclear and may increase pneumonia risk. This highlights the importance of procedure-specific evaluations. Further randomized trials are needed to clarify SDD’s risks and benefits in gastric cancer surgery, investigate mechanisms such as the gut-lung axis, and identify which patient groups may benefit or be adversely affected.

## Conclusion

Perioperative SDD did not reduce major infectious complications after open gastrectomy and was associated with a higher incidence of pneumonia. Microbiological findings further indicated shifts toward enterobacteriaceae and enterococci and the emergence of resistant isolates. These results suggest that SDD should not be routinely applied in gastric cancer surgery and warrant further prospective validation.

## Supplementary Information

Below is the link to the electronic supplementary material.

Supplementary Material 1

## Data Availability

The datasets generated and analyzed during the current study are available from the corresponding author on reasonable request.

## References

[1] Sung H, Ferlay J, Siegel RL et al (2021) Global cancer statistics 2020: GLOBOCAN estimates of incidence and mortality. CA Cancer J Clin 71:209–249. 10.3322/caac.21660 33538338 10.3322/caac.21660

[2] Smyth EC, Nilsson M, Grabsch HI, van Grieken NCT, Lordick F (2020) Gastric cancer. Lancet 396:635–648. 10.1016/S0140-6736(20)31288-5 32861308 10.1016/S0140-6736(20)31288-5

[3] Wang S, Xu L, Wang Q et al (2019) Postoperative complications and prognosis after radical gastrectomy for gastric cancer: a systematic review and meta-analysis. World J Surg Oncol 17:52. 10.1186/s12957-019-1593-9 30885211 10.1186/s12957-019-1593-9 PMC6423865

[4] Tsujimoto H, Ichikura T, Ono S et al (2009) Impact of postoperative infection on long-term survival after potentially curative resection for gastric cancer. Ann Surg Oncol 16:311–318. 10.1245/s10434-008-0249-8 19037699 10.1245/s10434-008-0249-8

[5] Yu J, Hu J, Huang C et al (2013) Impact of age and comorbidity on postoperative complications in patients with advanced gastric cancer after laparoscopic D2 gastrectomy. Eur J Surg Oncol 39:1144–1149. 10.1016/j.ejso.2013.06.021 23850088 10.1016/j.ejso.2013.06.021

[6] Inokuchi M, Kojima K, Kato K, Sugita H, Sugihara K (2014) Risk factors for postoperative pulmonary complications after gastrectomy for gastric cancer. Surg Infect (Larchmt) 15:314–321. 10.1089/sur.2013.031 24796353 10.1089/sur.2013.031

[7] Park DJ, Lee HJ, Kim HH et al (2005) Predictors of operative morbidity and mortality in gastric cancer surgery. Br J Surg 92:1099–1102. 10.1002/bjs.4952 15931657 10.1002/bjs.4952

[8] Tokunaga M, Kurokawa Y, Machida R et al (2021) Impact of postoperative complications on survival outcomes in patients with gastric cancer: exploratory analysis of a randomized controlled JCOG1001 trial. Gastric Cancer 24:214–223. 10.1007/s10120-020-01102-3 32601909 10.1007/s10120-020-01102-3

[9] Pang HY, Zhao LY, Wang H et al (2021) Impact of type of postoperative complications on long-term survival of gastric cancer patients: results from a high-volume institution in China. Front Oncol 11:587309. 10.3389/fonc.2021.587309 34707984 10.3389/fonc.2021.587309 PMC8542852

[10] Yuan P, Wu Z, Li Z et al (2019) Impact of postoperative major complications on long-term survival after radical resection of gastric cancer. BMC Cancer 19:833. 10.1186/s12885-019-6024-3 31443699 10.1186/s12885-019-6024-3 PMC6708212

[11] Miki Y, Makuuchi R, Tokunaga M et al (2016) Risk factors for postoperative pneumonia after gastrectomy for gastric cancer. Surg Today 46:552–556. 10.1007/s00595-015-1201-8 26077287 10.1007/s00595-015-1201-8

[12] Eto K, Yoshida N, Iwatsuki M et al (2022) Impact of type of gastrectomy on death from pneumonia Inelderly gastric cancer patients. World J Surg 46:425–432. 10.1007/s00268-021-06352-5 34748057 10.1007/s00268-021-06352-5

[13] Makuuchi R, Irino T, Tanizawa Y, Bando E, Kawamura T, Terashima M (2019) Esophagojejunal anastomotic leakage following gastrectomy for gastric cancer. Surg Today 49:187–196. 10.1007/s00595-018-1726-8 30317492 10.1007/s00595-018-1726-8

[14] Bracale U, Peltrini R, De Luca M et al (2022) Predictive factors for anastomotic leakage after laparoscopic and open total gastrectomy: a systematic review. J Clin Med 11:5022. 10.3390/jcm11175022 36078954 10.3390/jcm11175022 PMC9457286

[15] Alverdy JC, Hyoju SK, Weigerinck M, Gilbert JA (2017) The gut Microbiome and the mechanism of surgical infection. Br J Surg 104:e14–e23. 10.1002/bjs.10405 28121030 10.1002/bjs.10405 PMC7938814

[16] Schardey J, von Ahnen T, Schardey E et al (2022) Antibiotic bowel decontamination in Gastrointestinal surgery: a single-center 20 years’ experience. Front Surg 9:874223. 10.3389/fsurg.2022.874223 35651691 10.3389/fsurg.2022.874223 PMC9150795

[17] Schmitt FCF, Brenner T, Uhle F et al (2019) Gut Microbiome patterns correlate with higher postoperative complication rates after pancreatic surgery. BMC Microbiol 19:42. 10.1186/s12866-019-1399-5 30777006 10.1186/s12866-019-1399-5 PMC6379976

[18] Bogner A, Stracke M, Bork U et al (2022) Selective decontamination of the digestive tract in colorectal surgery reduces anastomotic leakage and costs: a propensity score analysis. Langenbecks Arch Surg 407:2441–2452. 10.1007/s00423-022-02540-6 35551468 10.1007/s00423-022-02540-6 PMC9468075

[19] Scheufele F, Schirren R, Friess H, Reim D (2020) Selective decontamination of the digestive tract in upper Gastrointestinal surgery: systematic review with meta-analysis. BJS Open 4:932–942. 10.1002/bjs5.50332 10.1002/bjs5.50332 PMC7709368 32749070

[20] Radulova-Mauersberger O, Oehme F, Doerell A et al (2022) Selective decontamination of the digestive tract in pancreatic head resections: a propensity score-matched analysis. J Clin Med 12:250. 10.3390/jcm12010250 36615050 10.3390/jcm12010250 PMC9820838

[21] Mibelli N, Oehme F, Radulova-Mauersberger O et al (2024) Bacterial shift and resistance pattern in pancreatic head resections after selective decontamination. J Gastrointest Surg 28:1844–1852. 10.1016/j.gassur.2024.08.030 39241947 10.1016/j.gassur.2024.08.030

[22] Pfeifer C, Noll S, Gerecke H et al (2017) A stability study of amphotericin B, colistin and tobramycin in a hydrophilic suspension. Eur J Hosp Pharm 24:235–241. 10.1136/ejhpharm-2016-000931 31156945 10.1136/ejhpharm-2016-000931 PMC6451449

[23] Mortensen K, Nilsson M, Slim K et al (2014) Consensus guidelines for enhanced recovery after gastrectomy: enhanced recovery after surgery (ERAS^®^) society recommendations. Br J Surg 101:1209–1229. 10.1002/bjs.9582 25047143 10.1002/bjs.9582

[24] Dindo D, Demartines N, Clavien PA (2004) Classification of surgical complications: a new proposal with evaluation in a cohort of 6336 patients and results of a survey. Ann Surg 240:205–213. 10.1097/01.sla.0000133083.54934.ae 15273542 10.1097/01.sla.0000133083.54934.ae PMC1360123

[25] Kalil AC, Metersky ML, Klompas M et al (2016) Management of adults with hospital-acquired and ventilator-associated pneumonia: 2016 clinical practice guidelines. Clin Infect Dis 63:e61–e111. 10.1093/cid/ciw353 27418577 10.1093/cid/ciw353 PMC4981759

[26] Brown RL, Sequeira RP, Clarke TB (2017) The microbiota protects against respiratory infection via M-CSF signaling. Nat Commun 8:1512. 10.1038/s41467-017-01803-x 29142211 10.1038/s41467-017-01803-x PMC5688119

[27] Fan S, Jiang H, Xu Q et al (2025) Risk factors for pneumonia after radical gastrectomy for gastric cancer: a systematic review and meta-analysis. BMC Cancer 25:840. 10.1186/s12885-025-14149-1 40336054 10.1186/s12885-025-14149-1 PMC12060482

[28] Reuvers JRD, Gaikhorst E, Ben ÂJ et al (2023) Cost-effectiveness of selective decontamination of the digestive tract to decrease infectious complications in colorectal cancer surgery. Eur J Surg Oncol 49:107116. 10.1016/j.ejso.2023.107116 37907018 10.1016/j.ejso.2023.107116

[29] Morris MS, Graham LA, Chu DI et al (2015) Oral antibiotic bowel Preparation significantly reduces surgical site infection rates and readmission in elective colorectal surgery. Ann Surg 261:1034–1040. 10.1097/SLA.0000000000001125 25607761 10.1097/SLA.0000000000001125

[30] Humphreys H, Winter R, Pick A (1992) The effect of selective decontamination of the digestive tract on Gastrointestinal enterococcal colonization in ITU patients. Intensive Care Med 18:459–463. 10.1007/BF01708581 1289369 10.1007/BF01708581

[31] Buelow E, Gonzalez TB, Versluis D et al (2014) Effects of selective digestive decontamination (SDD) on the gut resistome. J Antimicrob Chemother 69:2215–2223. 10.1093/jac/dku092 24710024 10.1093/jac/dku092

[32] Oostdijk EA, de Smet AM, Blok HE et al (2010) Ecological effects of selective decontamination on resistant gram-negative bacterial colonization. Am J Respir Crit Care Med 181:452–457. 10.1164/rccm.200908-1210OC 19965807 10.1164/rccm.200908-1210OC

