# Correction to: Perioperative selective decontamination of the digestive tract does not improve postoperative infectious complications after gastrectomy: a propensity score-matched analysis

**DOI:** 10.1007/s00423-026-03991-x

**Published:** 2026-02-07

**Authors:** Jasmin Hasanovic, Floris Berg, Christian Teske, Marius Distler, Jürgen Weitz, Daniel E. Stange, Felix Merboth

**Affiliations:** 1 Department of Visceral, Thoracic, and Vascular Surgery, University Hospital Carl Gustav Carus, Technische Universität Dresden, Fetscherstrasse 74, 01307 Dresden, Germany; 2 National Center for Tumor Diseases (NCT/UCC), Dresden, Germany; 3 German CancerResearch Center (DKFZ), Heidelberg, Germany; 4 University Hospital and Faculty of Medicine Carl Gustav Carus, Technische Universität Dresden, Dresden, Germany; 5 Helmholtz-Zentrum Dresden-Rossendorf (HZDR), Dresden, Germany

**Correction to: Langenbeck’s Archives of Surgery (2026)**

10.1007/s00423-026-03974-y

The original version of this article unfortunately contained an error introduced during the production process. The author’s name, ‘Jasmin Hasanovic,’ appeared as both the first and last author. ‘Jasmin Hasanovic’ should be listed as the first author, and ‘Felix Merboth’ should be listed as the last author.

The original article has been corrected.

