# Supplementary material for: Perioperative selective decontamination of the digestive tract does not improve postoperative infectious complications after gastrectomy: a propensity score-matched analysis
Source: Langenbecks Arch Surg. 2026 Jan 22;411(1):73. doi: 10.1007/s00423-026-03974-y (PMC12855432; doi:10.1007/s00423-026-03974-y)
Supplement: Supplementary file 1 — Supplementary Material 1 [file 423_2026_3974_MOESM1_ESM.docx]

**Supplementary Tables and Figures**

**Supplementary Table 1: Standardized mean differences (unmatched and matched cohorts)**

| Variable | matched | unmatched |
| --- | --- | --- |
| Age | 0,023 | 0,057 |
| Sex | 0,121 | 0,025 |
| BMI | 0,084 | 0,121 |
| ASA | 0,056 | 0,314 |
| Alcohol | 0,000 | 0,035 |
| Smoking | 0,198 | 0,285 |
| Diabetes mellitus | 0,122 | 0,208 |
| UICC stage | 0,017 | 0,119 |
| Neoadjuvant treatment | 0,033 | 0,141 |
| Surgical procedure | 0,065 | 0,087 |

**Supplementary Figure 1: Love plot**


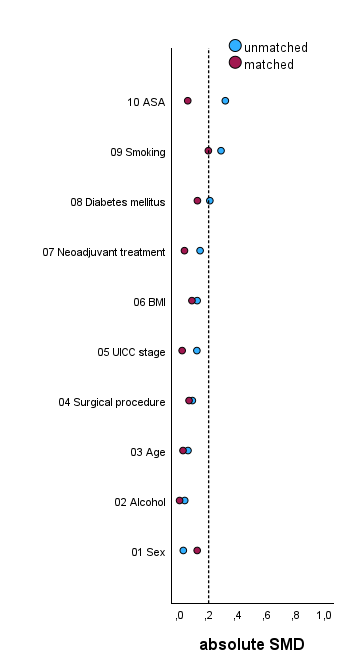


**Supplementary Table 2: Patient Characteristics Prior to Propensity Score Matching**

| Variable | SDD (n = 56) | No SDD (n = 85) | p-value |
| --- | --- | --- | --- |
| Age, years (median, IQR) | 70.6 (61.9–78.1) | 68.8 (59.7–75.9) | 0.624 |
| Male gender (n, %) | 46 (81.6) | 69 (81.2) | 0.885 |
| BMI (median, IQR) | 25.8 (22.7–28.8) | 25.4 (22.9–28.4) | 0.744 |
| ASA II (n, %) | 13 (23.2) | 26 (30.6) | 0.290 |
| ASA III (n, %) | 39 (69.6) | 53 (62.4) | 0.290 |
| ASA IV (n, %) | 4 (7.1) | 3 (3.5) | 0.290 |
| Alcohol use (n, %) | 5 (8.2) | 7 (8.2) | 1.000 |
| Smoking (n, %) | 13 (23.2) | 31 (36.5) | 0.096 |
| Diabetes mellitus (n, %) | 23 (41.1) | 26 (30.6) | 0.201 |
| Neoadjuvant chemotherapy (n, %) | 27 (48.2) | 49 (57.6) | 0.546 |
| Neoadjuvant radiochemotherapy (n, %) | 3 (5.4) | 4 (4.7) | 0.546 |
| No neoadjuvant therapy (n, %) | 26 (46.4) | 32 (37.6) | 0.546 |

Note: Values are shown as n (%) unless otherwise indicated.

SDD = selective decontamination of the digestive tract; BMI = Body mass index

**Supplementary Table 3: Operative Characteristics Prior to Propensity Score Matching**

| Variable | SDD (n = 56) | No SDD (n = 85) | p-value |
| --- | --- | --- | --- |
| Total gastrectomy (n, %) | 15 (26.8) | 26 (30.6) | 0.627 |
| Extended transhiatal gastrectomy (n, %) | 41 (73.2) | 59 (69.4) | 0.627 |
| R0 resection (n, %) | 50 (88.9) | 76 (89.4) | 0.981 |
| R1 resection (n, %) | 6 (10.7) | 9 (10.6) | 0.981 |
| Lymph nodes removed (median, IQR) | 23.5 (18.5 – 31) | 27 (22 – 34) | 0.288 |
| Positive lymph nodes (median, IQR) | 1 (0 – 1.5) | 1 (0 - 6) | 0.944 |
| Blood loss (mL, median, IQR) | 500 (300 – 600) | 600 (400 – 700) | 0.166 |

Note: Values are shown as n (%) unless otherwise indicated.

SDD = selective decontamination of the digestive tract.

**Supplementary Table 4: Postoperative Antibiotic Therapy**

| Variable | SDD (n = 54) | No SDD (n = 54) | p-value |
| --- | --- | --- | --- |
| Antibiotic Therapy received (n, %) | 12 (22.2) | 7 (13) | 0.206 |
| First line Antibiotics |  |  | 0.692 |
| Penicillin (n, %) | 1 (8.3) | 0 (0) |  |
| Piperacillin/Tazobactam (n, %) | 10 (84.2) | 6 (85.7) |  |
| Meropenem (n, %) | 1 (8.3) | 1 (14.3) |  |
| Second line Antibiotics |  |  | 0.753 |
| Piperacillin/Tazobactam (n, %) | 1 (20) | 0 (0) |  |
| Meropenem (n, %) | 2 (40) | 1 (100) |  |
| Linezolid (n, %) | 1 (20) | 0 (0) |  |
| Daptoomycin (n, %) | 1 (20) | 0 (0) |  |
| Length of Antibiotic Therapy (day, median, IQR) | 15 (7 – 25) | 8 (7 – 10) | 0.306 |

Note: Values are shown as n (%) unless otherwise indicated.

SDD = selective decontamination of the digestive tract.
